# Boundary Layer Control with a Plasma Actuator Utilizing a Large GND Mesh Electrode and Two HV Electrode Configurations †

**DOI:** 10.3390/s25010105

**Published:** 2024-12-27

**Authors:** Ernest Gnapowski, Sebastian Gnapowski, Paweł Tomiło

**Affiliations:** 1 Faculty of Mathematics and Information Technology, Lublin University of Technology, 20-618 Lublin, Poland; sgnapowski@wp.pl; 2 Faculty of Management, Lublin University of Technology, 20-618 Lublin, Poland; p.tomilo@pollub.pl

**Keywords:** plasma actuator, flow control, wing airfoil, wind tunnel, mesh electrodes, DBD

## Abstract

This article presents the results of experimental studies on the influence of the geometry of high-voltage plasma actuator electrodes on the change in flow in the boundary layer and their influence on the change in the lift coefficient. The plasma actuator used in the described experimental studies has a completely different structure. The experimental model of the plasma actuator uses a large mesh ground electrode and different geometries of the high-voltage electrodes, namely copper solid electrodes and mesh electrodes (the use of mesh electrodes, large GND and HV is a new solution). The plasma actuator was placed directly on the surface of the wing model with the SD 7003 profile. The wing model with the plasma actuator was placed in the wind tunnel. All experimental tests carried out were carried out for various configurations. The DBD plasma actuator was powered by a high-voltage power supply with a voltage range from *V_p_* = 7.5–15 kV. The use of a high-voltage mesh electrode allowed for an increase in the lift coefficient (C_L_) for the angle of attack *α* = 5 degrees and the air flow velocity in the range from *V* = 5 m/s to 20 m/s, while the use of copper electrodes HV with the plasma actuator turned off and on, were very small (close to zero). The experimental studies were conducted for Reynolds numbers in the range of Re = 87,985–351,939.

## 1. Introduction

A crucial aspect in aircraft design is ensuring high levels of safety for both passengers and the aircraft itself, being the reason why experimental tests are conducted on various components including landing gear [1,2,3,4], wings and their structures [5], turbine blades [6], and other elements [7]. Plasma actuators represent a relatively new technological solution aimed at enhancing flight safety. Many experimental studies worldwide focus on improving the efficiency of DBD plasma actuators, as their implementation contributes to increasing lift and enhancing safety. Plasma actuators allow the control of the boundary layer by DBD discharges [8,9]. The design of the DBD (Dielectric Barrier Discharge) plasma actuator is simple, as it does not involve moving parts like rods, valves, cylinders, or gears. The use of plasma actuators in aviation does not complicate wing design. The typical plasma actuator configuration used in research consists of solid electrodes (HV and GND) in the form of two flat copper strips separated by a dielectric in an asymmetric electrode setup. The dielectric serves as protection against spark discharges, which can lead to localized temperature spikes [10,11]. The first electrode, grounded (GND), is fully covered by the dielectric, while the second high-voltage (HV) electrode is exposed to the airflow.

**Figure 1:**
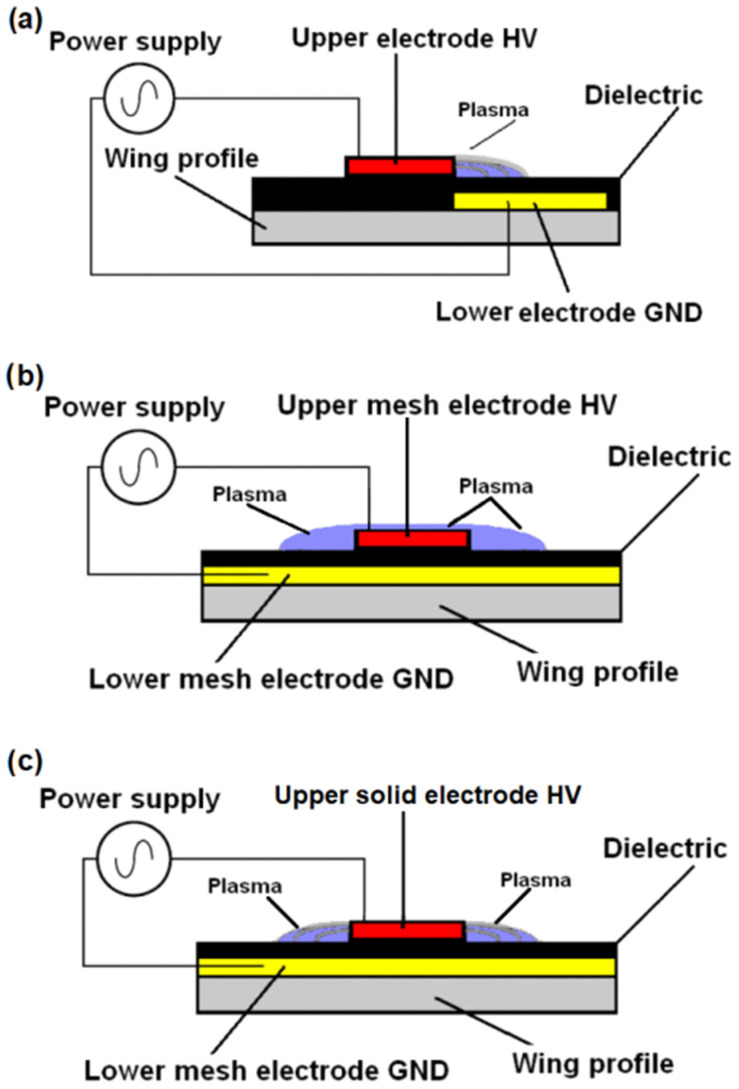
Configuration of electrodes in plasma actuators: (**a**) classic asymmetric system with two copper electrodes, (**b**) configuration with two mesh electrodes, (**c**) configuration with grounded mesh electrode and solid copper HV electrode.

The primary mechanism through which the DBD plasma actuator influences airflow is the generation of an “ion wind”, as discussed in numerous studies [9,10]. The operation of DBD plasma actuators relies on air ionization, which alters the potential of gas molecules and results in elastic collisions between migrating charged molecules and neutral gas molecules, increasing their energy and creating an “electric wind” near the surface of the airfoil [12,13,14,15]. Most research focuses on altering the shape of solid high-voltage copper electrodes to rectangular, oval, sawtooth, or corrugated forms. Modifying the shape of the HV electrode increases the discharge area of the plasma actuator (asymmetric configuration with two copper electrodes, Figure 1a) [16,17], producing a stronger “ion wind”, which improves airflow circulation in the boundary layer. Numerous experimental studies involving plasma actuators use power supply frequencies ranging from several hundred Hz to several kHz [18,19,20,21]. The range of applied voltages during experimental tests depends on the dielectric used [22,23,24,25] and is usually from a few to several dozen kV. It should be noted that the discharge frequency has a significant impact on the performance and power of the discharges. The efficiency of a 50 Hz system cannot be compared with that of a 2 kHz system, as the changes are not linear. The authors selected 50 Hz based on the studies of S. Okazaki [26], which confirmed the possibility of achieving homogeneous discharges at this frequency. Homogeneous discharges prevent local temperature increases and a decrease in system efficiency.

The manuscript describes a new plasma actuator design intended to control airflow in the boundary layer. Tests were conducted on a wing model with an SD 7003 airfoil for angles of attack between α = 5 and 15 degrees and air velocities from V = 5 to 20 m/s. The new plasma actuator design features a large, grounded mesh electrode covering 70% of the upper surface of the wing model (the use of large copper electrodes GND and HV is a new solution). The upper surface of the wing is completely covered with four layers of dielectric, separating the two electrodes. A high-voltage electrode was placed directly on the dielectric surface. Two HV electrode geometries were used in the experimental studies: the first series of tests used a solid oblique copper HV electrode, while the second series utilized an HV mesh electrode.

The expected effect of using oblique copper electrodes during experimental research is the enhancement of the vortex formation effect by combining the oblique arrangement of the electrodes similarly to the VG vortex generators setting and the plasma actuator effect.

- Another research goal was to verify the expected high efficiency of the plasma actu-ator by using a large GND mesh electrode, which allows discharges to form at both edges of the copper HV electrode, and in the second case, directly through the HV mesh electrode.
- The efficiency of the plasma actuator powered at a frequency of 50 Hz was evaluated during the experiments.
- It was also checked whether the frequency of 50 Hz is optimal for powering the tested plasma actuator.
- The primary objective of the wind tunnel tests was to assess the effectiveness of the new plasma actuator design and determine the optimal geometry for the HV electrode in the DBD system.
- The experimental tests also served to verify the assumptions related to the oblique arrangement of the copper electrodes.

The choice of comparing oblique HV electrodes and mesh electrodes was driven by the need to determine the impact of an oblique electrode configuration at a 15-degree angle (as in vortex generators) on the operation of the plasma actuator, and to compare it with a configuration utilizing a mesh HV electrode. There are no known reports in the literature of such experiments being conducted, as the configuration with the mesh GND electrode is a novel design. In the case of the copper HV electrode and the mesh GND electrode, discharges occur at both edges of the copper electrode, which was not possible in the classical configuration using an asymmetric arrangement of copper HV and GND electrodes.

The use of plasma actuators significantly reduces turbulence. This reduction in turbulence can lead to substantial energy savings required for movement [13,14,15]. Extensive research is being conducted on the application of plasma actuators on aircraft wing surfaces and in vehicles such as trucks to reduce turbulence and fuel consumption [26,27,28,29].

## 2. Materials and Methods

All experimental tests were conducted for various configurations of the angle of attack, ranging from *α* = 5 to 15 degrees, when changing every 5 degrees, and for air velocities between *V* = 5 and 20 m/s. It is important to note that the critical angle of attack for the tested SD 7003 airfoil profile is *α* = 11 degrees. Experimental tests with a plasma actuator were carried out at the State School of Higher Education in Chełm, in the Institute of Technical Sciences and Aviation. During the experiments, tests were conducted at an angle of attack α = 15 degrees higher than this critical angle. At angles exceeding *α* = 11 degrees, the SD 7003 airfoil loses all lift, resulting in stall. To enhance measurement accuracy, 10 measurements were taken for each configuration of attack angle and air velocity, both with the plasma actuator activated and deactivated. This approach allowed for a measurement uncertainty as low as 0.013 at an angle of attack of 15 degrees.

The tests were conducted in a wind tunnel (AeroLab tunnel) capable of regulating airspeed within the range of *V* = 4.5 to 65 m/s. The equipment installed in the tunnel enabled the measurement of forces acting on the wing during the experiments. During the experimental tests, the plasma actuator was powered by an autotransformer and a transformer increasing the voltage 230/10,000 V 50 Hz. Voltage and current discharges were recorded using a Keysight DSO-X 2012A oscilloscope (200 MHz, 2 GS/s) equipped with a Tektronix P6015A high-voltage probe and a Tektronix P2220 current probe with 1×/10× options.

**Figure 2:**
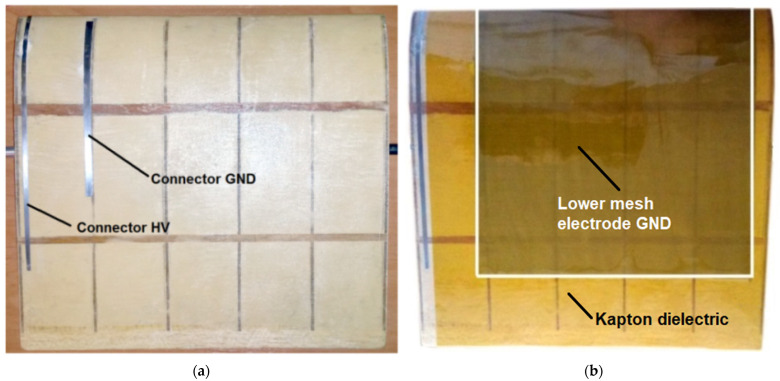
Construction and profile view of the SD7003 wing model and the wing model; (**a**) with a grounded electrode without dielectric, (**b**) covered with Kapton dielectric.

The wing model with SD7003 profiles, 250 mm wide and 250 mm long, is made of modeling plywood and balsa covered with fiberglass, as illustrated in Figure 2a. Two different electrode geometry configurations were tested during the experiments. In the first configuration, both the grounded electrode (GND) and the high-voltage (HV) electrode were made from AISI 304 stainless steel mesh (total mesh electrode thickness 0.1 mm). In the second configuration, only the GND electrode was made of mesh, while the HV electrode was a copper strip. In both configurations, the GND mesh electrode was positioned on the upper surface of the wing profile. The grounded mesh electrode was insulated with four layers of Kapton foil to prevent spark discharges, as shown in Figure 2b.

**Figure 3:**
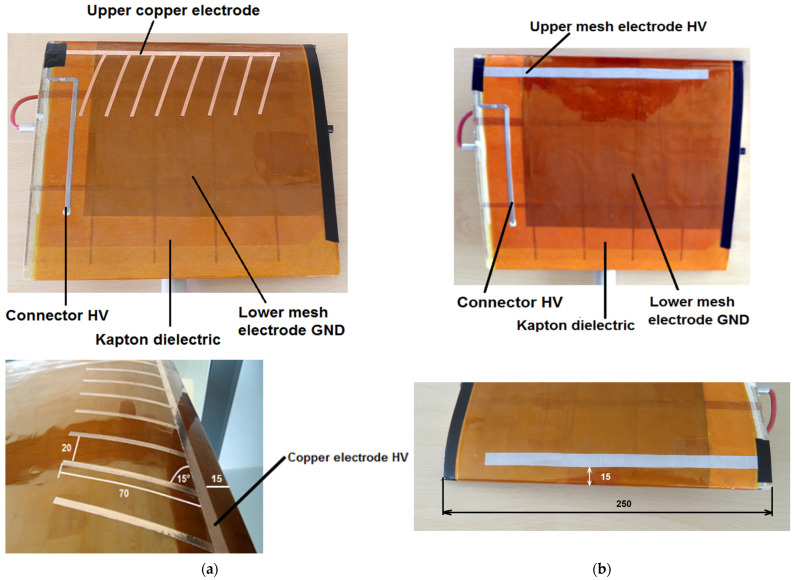
Configurations of the geometry of high-voltage electrodes used during experimental research; (**a**) copper electrodes, (**b**) mesh electrode.

A high-voltage mesh electrode with dimensions of 200 × 10 mm (mesh size 0.05 × 0.05 mm) was positioned 15 mm from the leading edge of the wing. This configuration enables the installation of a “large grounded electrode” and allows for the flexible movement of the upper high-voltage electrode along the HV connector, as illustrated in Figure 3a,b. In contrast to the commonly used plasma actuator with an asymmetrical electrode system, where the movement of the high-voltage electrode is not possible, this design offers greater flexibility in electrode positioning.

The design of the DBD system is relatively straightforward. It consists of two flat electrodes that are separated by a dielectric layer. The dielectric in DBD systems serves the important function of preventing localized temperature increases [9,10]. The most common configuration for a DBD plasma actuator is an asymmetrical electrode system, as shown in Figure 1a. This setup features two flat, parallel copper foil electrodes, separated by a thin dielectric layer, which are placed directly on the surface of the wing. In the operation of a plasma actuator with an asymmetrical electrode configuration, discharges occur only at one edge of the copper high-voltage (HV) electrode.

To perform the experimental studies described in this paper, two configurations were developed. In the first configuration, a mesh GND electrode and a copper tape HV electrode were used, while in the second configuration, both the GND and HV electrodes were mesh electrodes. Figure 1b illustrates the configuration and operation of the tested plasma actuator system using two mesh electrodes, while Figure 1c presents the system in a configuration featuring a grounded mesh electrode paired with a solid copper electrode. Unlike the solid electrodes typically used in plasma actuators, mesh electrodes are permeable to air and provide a larger discharge area. Experimental tests performed by the authors on plasma reactors operating at atmospheric pressure demonstrate that uniform, streamer-free discharges are achievable in DBD reactors with the use of mesh electrodes [7,24]. These properties of the mesh electrodes led the authors of this manuscript to apply them to the construction of a plasma actuator influencing the air flow in boundary layer of the aeronautical profile.

**Figure 4:**
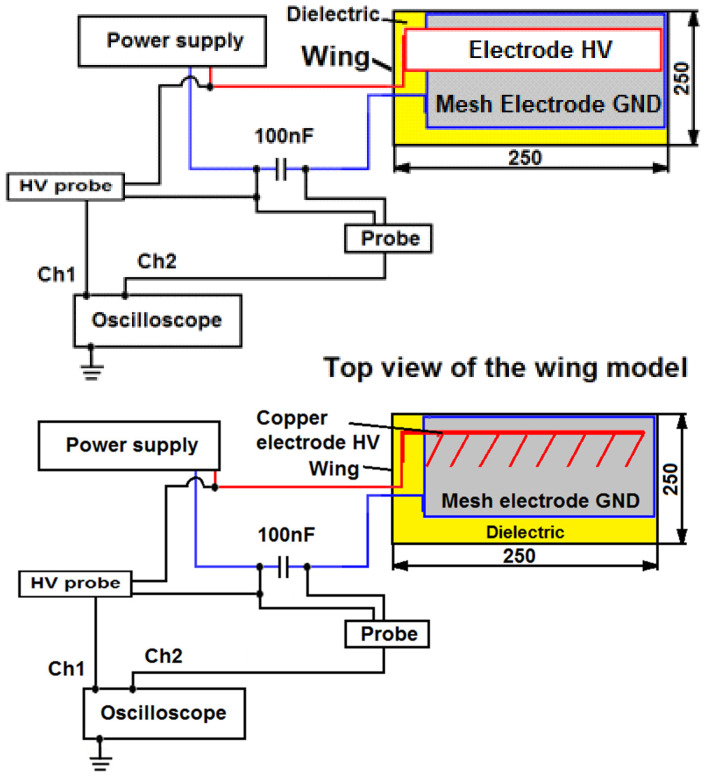
Diagrams of construction and operation of plasma actuators configuration of a system with two mesh electrodes and configuration of a grounded mesh electrode and a high-voltage copper tape, (red color—high voltage, blue color—GND, yellow color—Kapton dielectric, gray—GND electrode).

The measurement system setup connected to the wing model is depicted in Figure 4. The Phantom V2511 fast camera was used to record the tunnel images during the experimental tests.

The high-voltage copper electrode consists of 8 electrodes placed at angle *α =* 15 degrees connected together at one end, 15 mm from the leading edge, as shown in Figure 2a. The length of a single copper electrode is *l_e_* = 70 mm, with a width of 3 mm.

## 3. Results

The experimental tests in both configurations were conducted under identical conditions. During experimental tests, the discharge power varied between *P* = 1.1 W and 2.3 W, with a supply voltage ranging from *V_p_* = 7.5 kV to 15 kV. The power supply system for the plasma actuator operated at a frequency of 50 Hz. Several factors influenced the discharge power in the DBD system, including the frequency of the supply system, the type and thickness of the dielectric [10,30,31,32], the distance between electrodes, electrode geometry [22], and the type of gas used during the tests. The use of the AeroLab wind tunnel, equipped with measuring instruments, enabled the recording of forces acting on the wing throughout the experimental tests. A Keysight oscilloscope with equipment was used to measure DBD voltages and discharge currents, while the recorded Lissajous figure oscillograms facilitated the calculation of discharge power. During the wind tunnel tests, the forces acting on the wing model were recorded.

Results for the DBD plasma actuator configuration with two mesh electrodes (GND and HV): The first series of tests focused on a plasma actuator equipped with a large mesh GND electrode and a high-voltage mesh electrode. Table 1 presents the forces acting on the wing model for this two-mesh electrode plasma actuator configuration at an angle of attack of *α* = 5 degrees, comparing results with the plasma actuator turned on and off.

**Figure 5:**
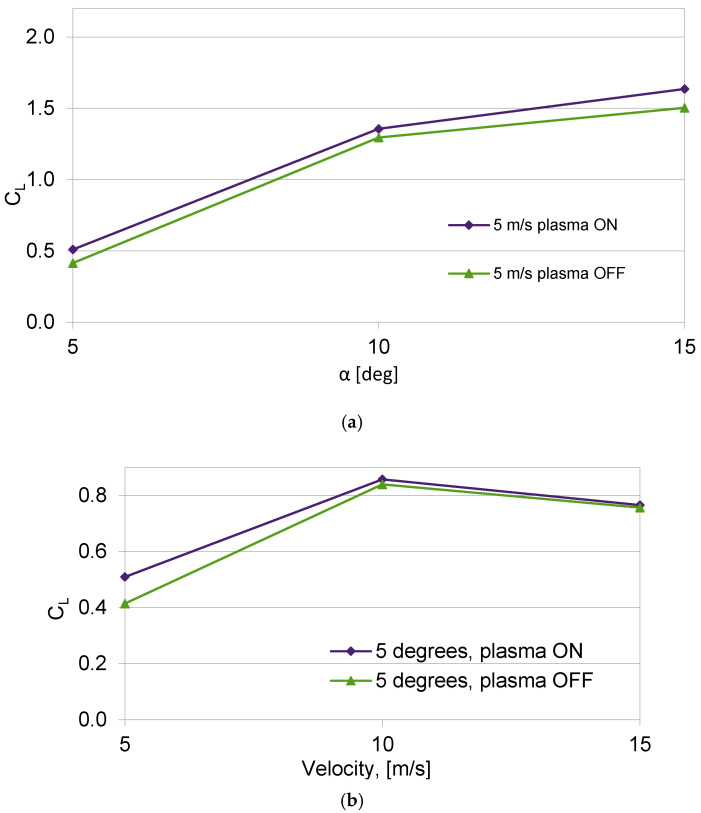
Changes in lift coefficient *C_L_* (**a**) for air flow velocity *V =* 5 m/s for angle of attack in the range from *α* = 5–15 degrees and (**b**) for angle of attack α = 5 degrees and air flow velocity *V =* 5–15 m/s; configuration with two mesh electrodes GND and HV.

**Figure 6:**
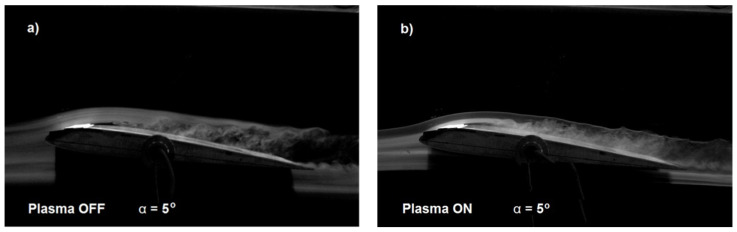

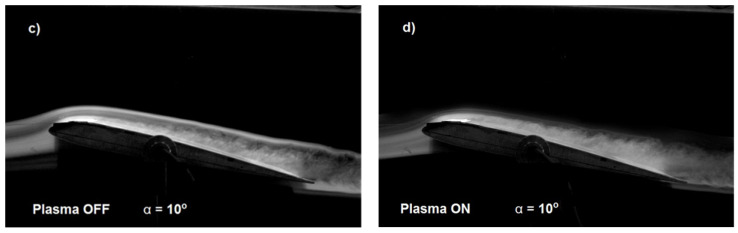
Tunnel photos taken for SD7003 wing profile with air flow velocity *V* = 5 m/s for angles of attack in the range from *α =* 5–10 degrees: plasma actuator turned off (**a**,**c**) and plasma actuator turned on (**b**,**d**).

**Figure 7:**
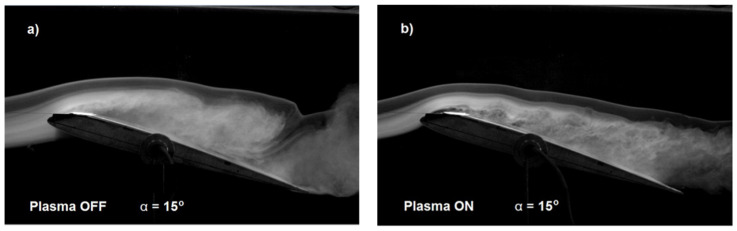
Tunnel photos taken for SD7003 wing profile with air flow velocity *V* = 5 m/s for angles of attack in the range from *α =* 15 degrees: plasma actuator turned off (**a**) and plasma actuator turned on (**b**).

One of the primary factors affecting changes in lift is the angle of attack. The changes in the lift coefficient (*C_L_*) for an air flow velocity of *V* = 5 m/s and angles of attack ranging from α = 5 to 15 degrees are depicted in Figure 5. The effectiveness of the newly configured plasma actuator is supported by high-speed camera images, as shown in Figure 6 and Figure 7. Another factor influencing the lift force is the increase in air flow velocity. As the velocity increases from *V* = 5 to 20 m/s for an angle of attack of *α* = 5 degrees, the lift coefficient decreases, as illustrated in Figure 5. The difference between the lift coefficient (*C_L_*) with the plasma actuator on and off diminishes as air flow velocity increases. The plasma actuator using two mesh electrodes demonstrates higher efficiency, which is further confirmed by the wind tunnel images shown in Figure 6 and Figure 7.

Numerous experimental studies have confirmed a decline in the efficiency of DBD systems as gas flow velocity increases, resulting in a decrease in ozone concentration (e.g., in ozone generators) [33,34,35]. Plasma actuators are DBD systems and when the gas flow velocity increases, their efficiency decreases [36,37,38,39]; increasing the frequency of the power supply system allows the partial remedying of this phenomenon.

### The Result Obtained for Plasma Actuator with Mesh Electrode GND and Oblique Copper Electrodes HV

Another series of experiments was conducted using a configuration with a large mesh ground (GND) electrode and high-voltage (HV) copper tape electrodes. The DBD plasma actuator shown in Figure 1c is a different design compared to the typical asymmetrical electrode geometry shown in Figure 1a, consisting of one grounded mesh electrode located on 70% of the upper wing surface and high-voltage copper electrodes. This configuration allows discharges to form along both edges of the copper HV electrode, increasing discharge efficiency. The total length of the copper electrodes is 760 mm. The use of copper electrodes does not allow the obtaining of discharges through of the electrodes surfaces like a mesh electrode. This setup represents a novel electrode geometry that combines copper electrodes with a mesh electrode. Table 2 provides data on the forces acting on the wing model at an angle of attack *α* = 5 degrees, with the plasma actuator turned on and off, in the oblique copper electrode HV configuration.

**Figure 8:**
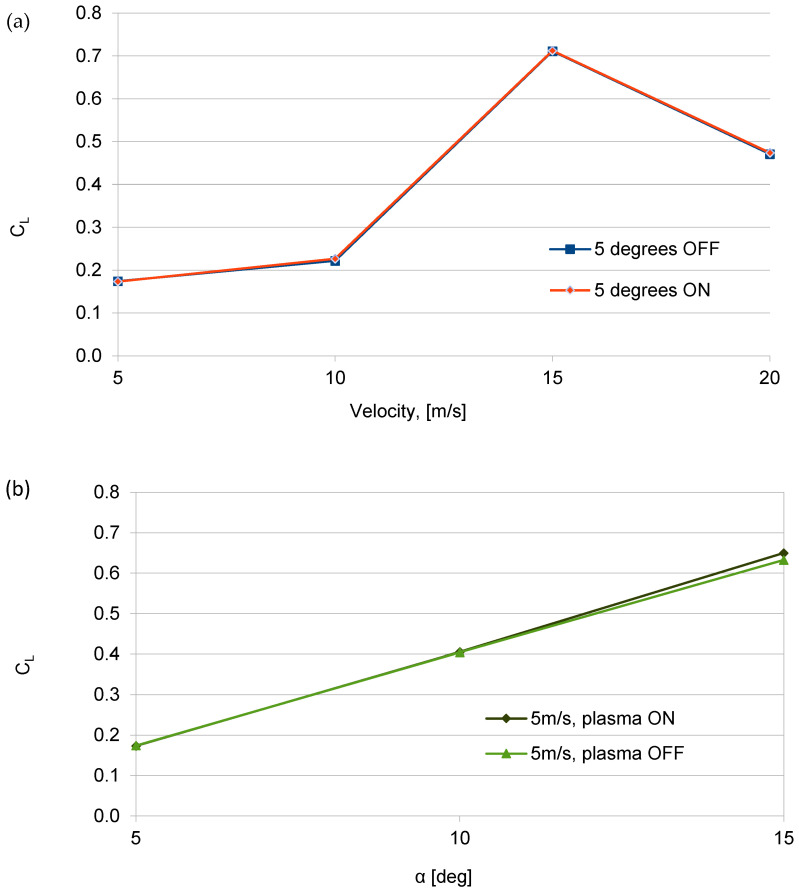
Changes in the lift coefficient (**a**) for angles of attack α = 5 degrees and air flow velocity in the range from *V* = 5 m/s to 20 m/s, with plasma actuator turned on and off, and (**b**) for air flow velocity *V* = 5 m/s for angles of attack in the range of *α* = 5–15 degrees, with plasma actuator turned on and off, with mesh electrode GND and oblique copper electrode HV.

To compare the efficiency of the plasma actuator with a mesh HV electrode and oblique copper HV electrodes, the same angle of attack (*α* = 5 degrees) and air flow velocity range were used as for the plasma actuator with two mesh electrodes (Figure 5). Changes in the lift coefficient depending on the air flow velocity are shown in Figure 8. The changes in *C_L_* for *α* = 5 degrees and air flow velocity in the range *V* = 5 to 20 m/s, with the plasma actuator turned on and off, are minimal (close to zero).

**Figure 9:**
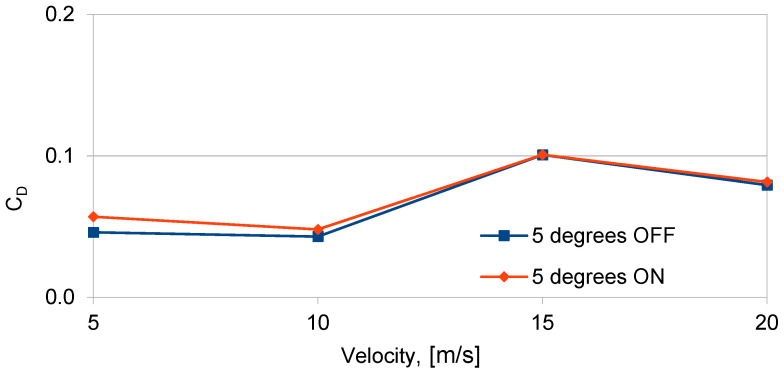
Changes in the drag coefficient *C_D_* depending on the air flow velocity for angle of attack *α* = 5, for configurations with plasma actuator turned off and on, with mesh electrode GND and oblique copper electrode HV.

In order to objectively compare the influence of the electrode geometry on the change in the lift force, a comparison was made of the influence of the angles of attack on the change in the lift coefficient (*C_L_*). Similarly to the analysis conducted for the plasma actuator with two mesh electrodes, this comparison was also performed for the copper electrode configuration. Figure 9 illustrates changes in the lift coefficient (*C_L_*) for air flow velocity *V* = 5 m/s and angles of attack ranging from *α* = 5 to 15 degrees for the plasma actuator with a copper HV electrode.

Data analysis and the graph in Figure 8 show only a small increase in the lift coefficient (*C_L_*), approximately 3%, at α = 15 degrees and air flow velocity *V* = 5 m/s when the plasma actuator is activated. The low efficiency of the plasma actuator results from the oblique geometry of the copper electrodes. Each of the eight electrodes generate a vortex on the two edges of each electrode. The oblique geometry of the electrodes causes the overlapping of subsequent vortexes arising on neighboring copper electrodes (generated vortices have mutually interlocked). This solution did not increase the lift force, as indicated by the shape and value changes in the lift coefficient shown in Figure 8, and it also increased drag. Changes in the drag coefficient (*C_D_*) as a function of air flow velocity for two angles of attack, *α* = 5 degrees and *α* = 10 degrees, with the plasma actuator on and off, are presented in Figure 9.

The analysis of the results obtained for the plasma actuator with oblique copper electrodes HV shows the increase in the drag coefficient, with a slight increase in the lift coefficient. The highest drag coefficient (*C_D_*) was observed for air flow velocities between *V* = 5 and 10 m/s at α = 5 degrees. With the decrease in the efficiency of the plasma actuator for angle of attack higher than 5 degrees and air flow velocity of more than *V* = 5 m/s, the generated drag decreased as the vortex generation intensity of the plasma actuator is reduced. Previous experimental studies have shown that the efficiency of plasma actuators decreases as the angle of attack and air flow velocity increase. This reduction in efficiency for the oblique HV copper electrode configuration is evident from the rise in drag. As the air flow velocity increases, the efficiency of the plasma actuator with the copper electrodes decreases, which can be seen after the decrease in the drag coefficient (*C_D_*) in Figure 9. The generated vortexes from individual oblique copper electrodes influenced each other by generating flow disturbances. An increase in disturbances increases the drag.

## 4. Conclusions

Experimental studies conducted in a wind tunnel on a plasma actuator in two configurations demonstrated changes in the lift coefficient due to the application of DBD discharges. The plasma actuator in the first configuration with a “large” mesh electrode (GND) and a high-voltage mesh electrode (HV), and in the configuration with a single “large” mesh electrode and an oblique solid copper electrode, are novel solutions not previously described in the literature. The wing model with the plasma actuator was subjected to wind tunnel tests at angles of attack ranging from *α* = 5 to 15 degrees and at four air flow velocities between *V* = 5 and 20 m/s. This allowed for the assessment of how these factors affect the plasma actuator’s efficiency. The results indicated that individual factors influence the actuator’s efficiency, particularly air flow velocity and angles of attack, which have a key role in forming the “ionic wind” and increasing lift force. Changes in the lift coefficient and the increase in turbulent flow as the angle of attack increases are illustrated in Figure 6 and Figure 7, caused by the separation point moving towards the leading edge.

The influence of air flow velocity on the lift coefficient is depicted in Figure 5 and Figure 8. The decrease in the lift coefficient with the increase in air flow velocity is the result of the low frequency of 50 Hz of the power supply system operation, which affects the efficiency and power of the plasma actuator. Based on the experimental results, it was concluded that the 50 Hz power supply frequency is too low for optimal operation of the plasma actuator in both the two mesh electrodes configuration and the copper electrodes configuration. The higher lift coefficient obtained for the plasma actuator with two mesh electrodes was achieved thanks to the use of a high-voltage mesh electrode. The use of a high-voltage mesh electrode allows for discharges to be obtained at both edges of the electrode and directly through the surface of the mesh electrode. The dual effect of the discharge being concentrated at the edge and directly through the surface of the mesh electrode allows for the generation of a stronger “ion wind”. The use of a large mesh electrode GND also allowed increased efficiency of the plasma actuator in the configuration with the HV copper electrode by obtaining discharges at both edges of the HV electrode (in this configuration it is not possible to obtain discharges directly through the electrode surface). The assumptions concerning the increase in the efficiency of the plasma actuator by the oblique mounting of the copper electrode, similarly to the VG vortex generators, did not generate the expected results, which is confirmed by the obtained results of experimental tests, presented in Figure 8 and Figure 9. Research carried out with copper electrodes showed that an important factor affecting the operation of the plasma actuator is the optimal arrangement of copper electrodes on the surface of the wing. The result obtained for the oblique copper electrodes showed an increase in drag with the plasma actuator switched on, this phenomenon is related to the overlap of vortexes and the generation of disturbances increasing the drag. It is necessary to optimize the plasma actuator system to maximize efficiency.

## Figures and Tables

**Table 1:** Configuration with two mesh electrodes, angle of attack α = 5 degrees.

V (m/s)	Normal Force (N)		Axial Force (N)	
	Plasma Actuator			
	OFF	ON	OFF	ON
5	0.40	0.48	0.03	0.09
10	3.33	3.40	0.16	0.18
15	6.57	6.65	0.33	0.36
20	7.40	7.61	0.34	0.40

**Table 2:** Configuration with oblique copper electrode HV, angle of attack *α* = 5 degrees.

V (m/s)	Normal Force (N)		Axial Force (N)	
	Plasma Actuator			
	OFF	ON	OFF	ON
5	0.17	0.17	0.02	0.02
10	0.85	0.88	0.07	0.08
15	6.18	6.19	0.33	0.33
20	7.29	7.33	0.56	0.56

## Data Availability

The original contributions presented in the study are included in the article, further inquiries can be directed to the corresponding author.
